# Sex Determination in the *Squalius alburnoides* Complex: An Initial Characterization of Sex Cascade Elements in the Context of a Hybrid Polyploid Genome

**DOI:** 10.1371/journal.pone.0006401

**Published:** 2009-07-28

**Authors:** Irene Pala, Manfred Schartl, Sólveig Thorsteinsdóttir, Maria Manuela Coelho

**Affiliations:** 1 Centro de Biologia Ambiental, Departamento de Biologia Animal, Faculdade de Ciências da Universidade de Lisboa, Lisbon, Portugal; 2 Physiologische Chemie I, Biozentrum, University of Würzburg, Würzburg, Germany; 3 Instituto Gulbenkian de Ciência, Oeiras, Portugal; Michigan State University, United States of America

## Abstract

**Background:**

Sex determination processes vary widely among different vertebrate taxa, but no group offers as much diversity for the study of the evolution of sex determination as teleost fish. However, the knowledge about sex determination gene cascades is scarce in this species-rich group and further difficulties arise when considering hybrid fish taxa, in which mechanisms exhibited by parental species are often disrupted. Even though hybridisation is frequent among teleosts, gene based approaches on sex determination have seldom been conducted in hybrid fish. The hybrid polyploid complex of *Squalius alburnoides* was used as a model to address this question.

**Methodology/Principal Findings:**

We have initiated the isolation and characterization of regulatory elements (*dmrt1*, *wt1*, *dax1* and *figla*) potentially involved in sex determination in *S. alburnoides* and in the parental species *S. pyrenaicus* and analysed their expression patterns by *in situ* hybridisation. In adults, an overall conservation in the cellular localization of the gene transcripts was observed between the hybrids and parental species. Some novel features emerged, such as *dmrt1* expression in adult ovaries, and the non-dimorphic expression of *figla*, an ovarian marker in other species, in gonads of both sexes in *S. alburnoides* and *S. pyrenaicus*. The potential contribution of each gene to the sex determination process was assessed based on the timing and location of expression. *Dmrt1* and *wt1* transcripts were found at early stages of male development in *S. alburnoides* and are most likely implicated in the process of gonad development.

**Conclusions/Significance:**

For the first time in the study of this hybrid complex, it was possible to directly compare the gene expression patterns between the bisexual parental species and the various hybrid forms, for an extended set of genes. The contribution of these genes to gonad integrity maintenance and functionality is apparently unaltered in the hybrids, suggesting that no abrupt shifts in gene expression occurred as a result of hybridisation.

## Introduction

The genetic basis of sex determination and gonad development processes has been intensively studied in several animal groups and strict regulatory networks have been revealed in birds and mammals [1]. In fish a wide spectrum of reproductive systems is present, ranging from hermaphroditism to gonochorism and a frequent switch between mechanisms as diverse as environmental or strictly genetic based sex determination can be observed [2]–[4]. Among non-model fish, groups that have arisen through hybridisation and polyploidy offer additional challenges, as they often present reproductive and sex distribution alterations that imply an even broader diversity of regulatory processes [5]. Polyploidisation processes, as postulated by Ohno [6], have been important drives for vertebrate evolution [7], [8] and a particular relevant basis underlying speciation and diversity of teleost fish [9]. However, little is known about how sex determination cascades are regulated in hybrid fish species. In fish, evolutionary proximity is no guarantee of similarity in sex determination mechanisms. Hybridisation, even between closely related species [10], frequently brings about an abrupt shift in the regulatory networks, as a consequence of the interaction between distinct parental gene hierarchies [11]. As an emblematic example of such a deviation, the *Squalius alburnoides* complex of hybrid cyprinid fish presents unique features that make it an interesting model to address the question of sex determination in a hybrid context. This complex is endemic from the Iberian Peninsula and resulted from interspecific hybridisation between females of *Squalius pyrenaicus* (P genome) and males of an unknown species related to *Anaecypris hispanica* (A genome). The complex includes individuals of various ploidy levels that intercross through highly diverse reproductive modes, ranging from clonal inheritance to normal meiosis, hybridogenesis or meiotic hybridogenesis (in which one genome is excluded from gamete formation) [12]. Sex ratio distribution is clearly altered in the *S. alburnoides* complex, with a strong bias towards triploid females of PAA genotype. The complex also includes a lineage of individuals of AA genotype, apparently composed exclusively of males [12]. The strong correlation between genomic constitution and sex is an interesting feature to explore in terms of the sex determination process in these fish. Until now, no sex chromosomes that would indicate a chromosomal based sex determination have been identified, although chromosome heteromorphism that would suggest a ZWfemale/ZZmale system has been reported for the maternal ancestor *S. pyrenaicus* [13]. Data from experimental crosses revealed that such a system could not fully explain sex determination in *S. alburnoides* and that in addition to female determinants on the W chromosome, a minimum of one non-W-linked gene would have to be expressed differently in hybrid and nonhybrid genome combinations to account for the results obtained. Moreover, the “strength” of these sex-determining factors might be variable depending on populations and parental species [14].

So far, only one sex determination gene (the anti-Müllerian hormone, *amh*) has been isolated in *S. alburnoides*, and its expression characterized both during development and in the adult [15]. To fully explore the possibility of a genetically based sex determination mechanism, an initial set of possible candidates has to be established. It has been shown that despite the variety of mechanisms, the components of sex determination cascades are apparently conserved throughout the whole vertebrate lineage [16]–[18], although their position in gene hierarchies and their interaction patterns can vary according to group. The study of an initial set of conserved genes in the *S. alburnoides* complex could then constitute the starting point towards a broader understanding of the impact of abrupt genomic remodeling processes such as hybridisation on gene hierarchies and more broadly elucidate their putative roles within the fish lineage.

The *Dmrt1* (*doublesex* and *mab-3* related transcription factor 1) gene is part of a gene family sharing a common zinc-finger DNA binding motif (the DM domain). The DM domain was initially identified in genes that occupy key positions in sex determination pathways of the fruit fly *Drosophila melanogaster* (the *doublesex* gene) [19] and in the nematode *Caenorhabditis elegans* (*mab-3* gene) [20], but *Dmrt1* homologues have been shown to be expressed mainly in the adult and developing gonads, and correlated to sex determination cascades in a number of species [21]. In humans, although acting downstream in the sex determination cascade, *DMRT1* has been implicated in some types of XY sex reversal [22]. In birds, its location on the Z chromosome [23] and the higher dosage in males [16], [24], [25] make it a very good candidate for the male sex determining gene. In fish, it has been implicated in male sex determination pathways in the Nile tilapia *Oreochromis niloticus* [26] and rainbow trout *Oncorhynchus mykiss* [27], and shown to contribute to both male and female gonad development in zebrafish [28]. Most prominently, in the medaka *Oryzias latipes*, a *dmrt1* duplicate on the Y chromosome (*dmrt1bY/DMY*) [29], [30] has been identified as the master male sex determination gene in this species.

The Wilm's tumor suppressor gene (*Wt1*) is a key regulator of urogenital development. The gene encodes a nuclear protein containing four zinc fingers, acting both as a transcription factor and in RNA processing. In non-mammalian vertebrates two isoforms occur, resulting from alternative splicing [31]. The most important functional difference within all isoforms results from the insertion of three additional amino acids (KTS) by splicing events: the WT1 (-KTS) isoform acts as a transcription factor, while WT1 (+KTS) is mainly involved in RNA processing [32], [33]. In the medaka, an additional splice variant of Wt1, *wt1a*_dE4, with differences in exon 4 is present in both +KTS and –KTS isoforms [34]. The involvement of *Wt1* in the differentiation of urogenital structures and the maintenance/proliferation of the cells of the bipotential gonad has been shown in mammals [35], in chicken [36], in reptiles [37] and amphibians [38]. *Wt1* orthologs have been isolated in telosts species, such as the medaka *O. latipes* [39], [34], zebrafish *D. rerio* [40], the Japanese eel *Anguilla japonica* [41] and the rainbow trout *O. mykiss* [42]. Considering its role in gonad differentiation a few possible transcriptional targets of *Wt1* have been identified, such as *Dax1*, *Sf1* and *Gata 4* [43], [44].

The third gene, *dax1*, is a member of the nuclear receptors superfamily, with a ligand-binding domain at the C-terminal and LxxLL motifs, which enable the binding and interaction with potential targets (reviewed in [45]). Dax1 acts as a transcriptional repressor of several genes involved in the development and steroidogenic activity of adrenal and gonadal structures, such as aromatase *cyp19* [46], anti-müllerian hormone *amh* [47], estrogen and androgen receptors [48], [49], and most notably was shown to inhibit Sf1-mediated transcriptional transactivation [50]. *Dax1* expression is normally restricted to specific tissues, namely the genital ridge and it is largely coexpressed with *Sf1*, antagonizing its cooperation with WT1 and GATA-4 in the regulation of AMH [51], [47]. *Dax1* orthologs have been isolated and studied in different groups such as birds [52], reptiles [37], [53] and amphibians [54]. In fish, *dax1* expression patterns have been investigated both regarding its function in adrenal development (in the zebrafish, *D. rerio*) [55] and its contribution to gonad development and differentiation in several species, such as the nile tilapia *O. niloticus* [56], the European sea bass *Dicentrarchus labrax* [57] and the medaka *O. latipes* [58].

The fourth gene that is reported to be involved in early sex differentiation of several groups is the Factor in the germline alpha (*Figla*), a germ cell specific basic helix-loop-helix transcription factor. This factor plays a key role in folliculogenesis, by coordinating the regulation of the zona pellucida genes [59]. Absence of *figla* expression has been shown to affect female, but not male fertility in mice, suggesting a critical role in female germline development [60].

In a system such as the *S. alburnoides* complex, in which no genomic information is available, our strategy was to isolate potential candidates for sex determination and assess, based on their conservation, functional characteristics and expression patterns whether they could have a role in the establishment of the phenotypic sex. In the present work we report the isolation of the *dmrt1*, *wt1*, *dax1* and *figla Squalius* orthologs, the characterization of their specific features with respect to potential functionality, and the description of their expression in the gonads of the bisexual species and hybrids by *in situ* hybridisation. Finally, we evaluated the potential for a more extensive analysis of their contribution for male or female gonad development in the *S. alburnoides* complex. This constitutes a first attempt of comparing gene expression patterns between fish hybrids and one of their parental species, in the pursuit of a better understanding how gene hierarchies underlying sex determination might be affected by the hybridisation process.

## Results

### Isolation of sexual development genes – structural and phylogenetic analysis

#### *dmrt1*

**Figure 1:**
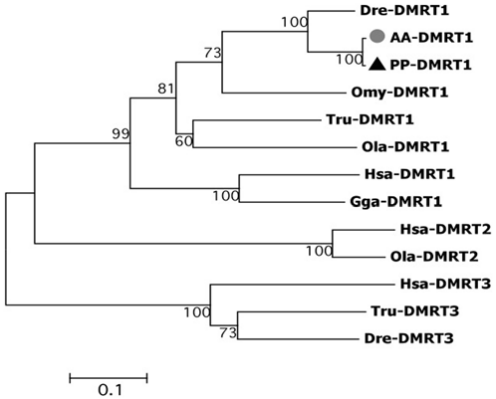
Unrooted Neighbour-Joining tree obtained among the DMRT1 family and other proteins that share the conserved DM domain. Dre-DMRT1- *Danio rerio*; Tru-DMRT1- *Takifugu rubripes*; Omy-DMRT1- *Oncorhynchus mykiss*; Ola-DMRT1- *Oryzias latipes*; Hsa-DMRT1- *Homo sapiens*; Gga-DMRT1- *Gallus gallus*; DMRT2-Hsa- *H. sapiens*; DMRT2-Ola- *O. latipes*; Hsa-DMRT3- *H. sapiens*; DMRT3-Tru- *T. rubripes*; DMRT3-Dre- *D. rerio*. Bootstrap values are shown above the branches. ▴ *S. pyrenaicus* (PP); • *S. alburnoides* (AA).

Amplification of a partial fragment of the *dmrt1* gene was performed using primers DMRT1- F1 D and DMRT1 zf-R3 and sequences from *S. pyrenaicus* (P genome) and *S. alburnoides* (A genome) were obtained (Table 1). Both fragments included the specific DM DNA binding domain that characterizes this class of proteins (Figure S1). The conservation of functional motifs, in comparison to other DMRT1 proteins (Figure S1) and the phylogenetic proximity to the Dmrt1 genes of other teleosts, revealed by the analysis of different proteins that share the conserved DM domain (Figure 1) confirmed the isolation of the Dmrt1 ortholog in *S. pyrenaicus* and *S. alburnoides*.

**Table 1:** Primer sequences (primers designed in the present work), references and GenBank accession numbers for each gene.

Gene	Primer	Sequence/Ref	Accession nr.
*dmrt1*	DMRT1- F1 D	Pala *et al.*, 2008b	EU199439
	DMRT1 zf-R3		EU199440
*wt1*	WT1 F1a	5′- ATGGGYTCWGAYGTKCGTGACC-3′	
	WT1 R1a	5′- GCMTAYCCTGGCTGCAACAAA -3′	
	WT1 F1b	5′- AGCTGCAGCACHCAGTAYC-3′	FJ587497
	WT1 R1b	5′- TTGSTCAKRTTKCTCTGRTGC-3′	
	WT1 F5	5′- CACTTCTCYGGACAGYTCA -3′	
*dax1*	DAX1 F1	5′- TCTGCGAGGATGGCTTACTT -3′	FJ587498
	DAX1 R1	5′- CATGGAGAGAGCGAGGAAGA -3′	FJ587499
*figla*	FIG F2	5′- CGAGATAAAGCTGTGAAGAGG-3′	FJ587500
	FIG R1	5′- CCTGACGTCATTGTGACCAG -3′	FJ587501

A two amino acid (Threonine and Aspargine) deletion followed by an amino acid substitution (Leucine by Isoleucine) was observed on the predicted sequence of *S. alburnoides* (A genome) compared to *S. pyrenaicus* (P genome), but no relevant differences in the secondary structure predicitions of the protein were found as a consequence of the genome-specific polymorphism.

#### *wt1*

**Figure 2:**
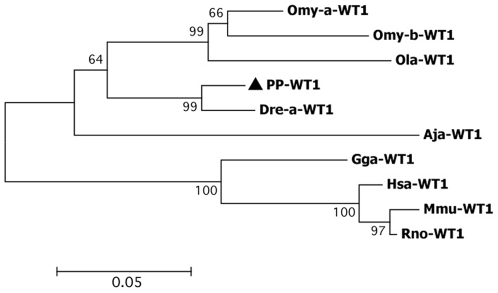
Unrooted Neighbour-Joining tree obtained among the teleost Wt1 proteins and other WT1 orthologs. Dre-a-WT1- *Danio rerio*, Omy-a-WT1; Omy-b-WT1- *Oncorhynchus mykiss*, Aja-WT1- *Anguilla japonica*; Ola-WT1- *Oryzias latipes*; Hsa-WT1- *Homo sapiens*, Gga-WT1- *Gallus gallus*, Mmu-WT1- *Mus musculus*, and Rno-WT1- *Rattus norvegicus*. Bootstrap values are shown above the branches. Bootstrap values are shown above the branches. ▴ *S. pyrenaicus* (PP).

Of all primer combinations tested, only the ones involving WT1-F1a and, WT1-R1a and WT1-R1b, respectively (Table 1) resulted in the production of sequence segments that could be identified as *wt1*. BLAST comparison revealed an overall similarity of all amplified fragments with WT1a transcripts of other teleosts. A larger PCR fragment was amplified with primers WT1 F5 and WT1-R1a (Table 2), using *S. pyrenaicus* gonad cDNA as template. The closer homology of our isolate to a-form Wt1 proteins of other telelosts was confirmed both in the phylogenetic tree comparing other Wt1 orthologs (Figure 2) and the alignments of the predicted amino acid sequence (Figure S2). Three distinct domains were identified in the *S. pyrenaicus* Wt1 isolate (Figure S2): a Wilm's tumour specific domain, and two (of the four) zinc finger DNA-binding motifs that characterize the C terminus of this transcription factor.

**Table 2:** Summary of amplification results for the three microsatellite loci (LCO3, LCO4, LCO5) in adult samples (a) and in the parents and progeny of the experimental cross (b): Species (*S. alburnoides* and *S. pyrenaicus*); Sex; n (number of the individual); P (ploidy); nr (number of offspring with the same allelic combination); alleles identified for each locus; and (G) genotype.

**a- Adult samples**							
**Species**	**Sex**	**P**	**n**	**LCO3**	**LCO4**	**LCO5**	**G**
*S.pyr*	M	2n	131	243^P^243^P^	238^ P^238^P^	137^P^143^p^	PP
*S.pyr*	M	2n	137	243^P^243^P^	240^P^240^P^	137^P^137^p^	PP
*S.pyr*	F	2n	132	243^P^243^P^	240^P^240^P^	137^P^143^p^	PP
*S.pyr*	F	2n	5	243^P^243^P^	238^ P^240^P^	137^P^137^p^	PP
*S.alb*	M	2n	106	247^A^247^A^	286^A^302^A^	131^A^131^A^	AA
*S.alb*	M	2n	110	247^A^247^A^	278^A^280^A^	131^A^131^A^	AA
*S.alb*	M	2n	85	243^P^247^A^	238^P^284^A^	131^A^143^P^	PA
*S.alb*	F	2n	86	243^P^247^A^	240^P^290^A^	131^A^137^P^	PA
*S.alb*	F	3n	90	243^P^247^A^249^A^	268^P^276^A^276^A^	131^A^131^A^143^P^	PAA
*S.alb*	F	3n	117	243^P^247^A^247^A^	238^P^282^A^302^A^	131^A^131^A^137^P^	PAA
**b- Embryos- Experimental cross**							
**Parents**							
**Species**	**Sex**	**P**		**LCO3**	**LCO4**	**LCO5**	**G**
*S.alb*	F	3n		243^P^247^A^	238^P^266^A^272^A^	131^A^137^P^	PAA
*S.alb*	M	2n		247^A^247^A^	274^A^286^A^	131^A^131^A^	AA
**Progeny**			**nr**				
*S.alb*		2n	3	247^A^247^A^	266^A^286^A^	131^A^131^A^	AA
*S.alb*		2n	4	247^A^247^A^	266^A^274^A^	131^A^131^A^	AA
*S.alb*		2n	1	247^A^247^A^	272^A^286^A^	131^A^131^A^	AA
*S.alb*		2n	1	247^A^247^A^	272^A^286^A^	131^A^131^A^	AA

#### *dax1*

**Figure 3:**
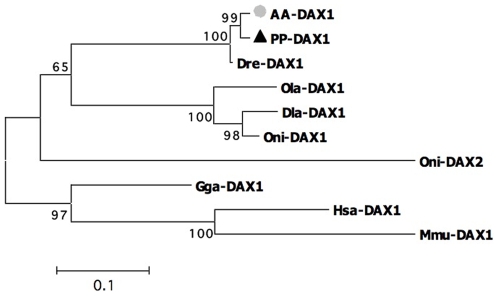
Unrooted Neighbour-Joining tree obtained among the DAX1 family. Dre-DAX1- *Danio rerio*, Dla-DAX1- *Dicentrarchus labrax*, Ola-DAX1- *Oryzias latipes*, Ony-DAX1- *Oreochromis niloticus*, Hsa-DAX1- *Homo sapiens*, Mmu-DAX1- *Mus musculus* and Gga-DAX1- *Gallus gallus*. Dax2 protein of *Oreochromis niloticus* (Oni-DAX2). Bootstrap values are shown above the branches. ▴ *S. pyrenaicus* (PP) ; • *S. alburnoides* (AA).

Fragments corresponding to a partial coding sequence of the *dax1* gene were amplified with primers DAX1-F1 and DAX1-R1 using gonad cDNA samples of *S. pyrenaicus* and *S. alburnoides* as templates (Table 1). Alignment of the predicted amino acid sequences revealed 96% identity with the Dax1 protein of zebrafish and 60-64% identity with Dax1 proteins of other teleosts (Figure S3). The phylogenetic reconstruction (Figure 3) revealed a close evolutionary proximity to the Dax1 protein of zebrafish, with both cyprinids grouping with the Dax1 group of proteins of other teleosts.

The domain prediction analysis confirmed that the *Squalius* isolates included functional regions that characterize Dax1 proteins and that are relevant for their role as transcription factors (Figure S3). The *Squalius dax1* gene encodes for two LxxLL motifs, an important feature for mediating protein-protein interactions and a typical characteristic of non-mammalian Dax1 proteins (as opposed to the four LxxLL motifs in mammals) [55]. Furthermore, a conserved ligand-binding domain, characteristic of hormone receptors was predicted within our isolate. Thus, although it corresponds to a partial coding sequence, the *dax1* fragment isolated from *S. alburnoides* and *S. pyrenaicus* includes all relevant features that characterize this family of proteins.

#### *figla*

**Figure 4:**
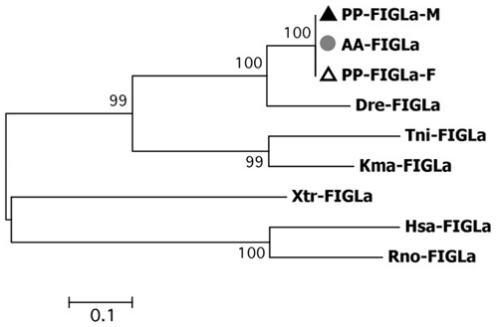
Unrooted Neighbour-Joining tree obtained among the FIGLA family of proteins. Dre-FIGLA- *Danio rerio*, Tni-FIGLA- *Tetraodon nigroviridis*, Kma-FIGLA- *Kryptolebias marmoratus*, Hsa- FIGLA- *Homo sapiens*, Rno-FIGLA- *Rattus norvegicus*, and Xtr FIGLA- *Xenopus (Silurana) tropicalis*. Bootstrap values are shown above the branches. ▴ *S. pyrenaicus* males (PP-M); ▵ females (PP-F) and • *S. alburnoides* (AA).

Fragments of approximately 300 bp corresponding to a putative *figla* product were obtained using *S. alburnoides* (AA) and *S.pyrenaicus* (PP) as templates. Successful amplification was obtained both with male and female gonad samples and no sequence differences were found according to sex. This is a surprising finding, as *figla* was expected to be an exclusive ovarian marker and the presence of a specific transcript of this gene was only expected in female gonads. The alignment of the predicted amino acid sequences (Figure S4) revealed a high identity with Figla proteins of several teleosts. A Helix-loop-helix domain was identified (Figure S4), which is a diagnostic feature of the Figla transcription factor, enabling the conversion of monomers to trans-activating dimmers. The phylogenetic analysis (Figure 4) revealed a preferential grouping with zebrafish Figla, and a closer evolutionary relatedness to other teleosts' proteins, thus confirming our isolate as a partial sequence of the *Squalius* Figla ortholog.

### Gene expression patterns

#### *dmrt1*

**Figure 5:**
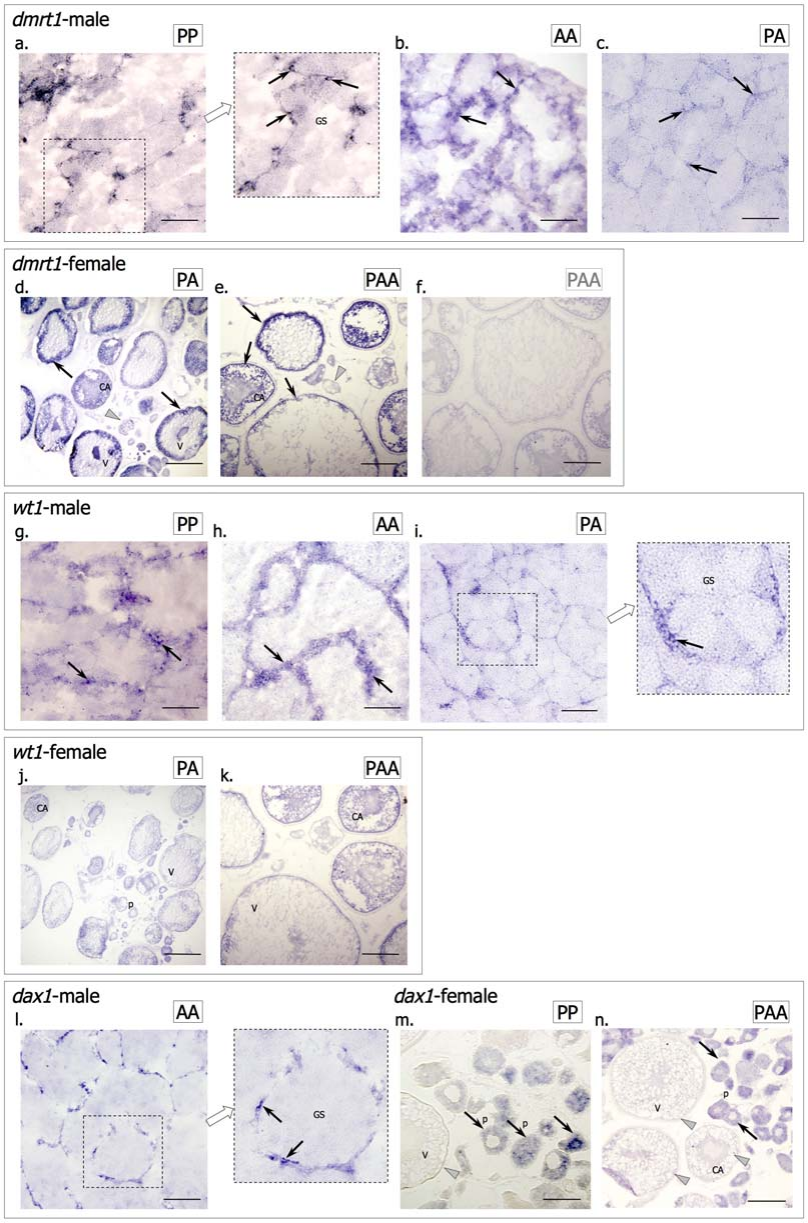
Expression patterns of candidate genes in the adult gonads *S. pyrenaicus* and *S. alburnoides*. *Dmrt1*: (a) *S. pyrenaicus* (PP genotype); (b) *S. alburnoides* (AA genotype) and (c) *S. alburnoides* (PA genotype) testis; (d) *S. alburnoides* (PA genotype) and *S. alburnoides* (PAA genotype) ovary with (e) antisense and (f) sense probes. *Wt1*: (g) *S. pyrenaicus* (PP genotype); (h) *S. alburnoides* (AA genotype) and (i) *S. alburnoides* (PA genotype) testis; (j) *S. alburnoides* (PA genotype) and (k) *S. alburnoides* (PAA genotype) ovary. *Dax1*: (l) *S. alburnoides* (AA genotype), (m) *S. pyrenaicus* (PP genotype) and (n) *S. alburnoides* (PAA genotype) ovary. Examples of areas with positive signal are indicated by black arrows; examples of negative cells are highlighted with grey arrowheads. Germ cells (GS), early perinuclear (P), cortical alveolar (CA) and vitellogenic (V) oocytes. Scale bar = 100 µm (a, b, e, f, g, h, k, l, m); scale bar = 200 µm (c, d, i, j, n).

Expression of *dmrt1* was observed in specific cellular locations in males of the bisexual ancestor *S. pyrenaicus* (PP) and both in nuclear non-hybrid males (AA) and diploid hybrids (PA) of *S. alburnoides* (Figure 5a, b and c). The location of *dmrt1* signal is observed in positions expected for Sertoli cells, as predicted previously from morphological comparison and gonad structural analysis [15]. Since the signal is more diffuse in AA gonads, we cannot exclude that *dmrt1* may also be expressed in some peripheral spermatogonia (Figure 5b). Otherwise, no differences were observed between hybrids and parental species in terms of the location of positive sites of *dmrt1* expression, although a comparatively stronger signal was usually obtained in PP and AA gonads (Figure 5a and b) compared to PA male hybrids (Figure 5c). *Dmrt1* expression was observed in adult ovaries of diploid (PA) and triploid (PAA) hybrid females of *S. alburnoides*. Expression was confined to the more developed cortical alveolar and yolk vesicle oocytes and was apparently absent from earlier stage oocytes (Figure 5d and e).

*Dmrt1* transcripts were detected early during male gonad development in the hybrids of *S. alburnoides*, suggesting a potential role already at these early stages. The presence of a *dmrt1* specific transcript was also detected by RT-PCR at early stages in *S. alburnoides* AA progeny and its expression in the presumptive region of gonad development was observed by in situ hybridisation, as early as 6 days after hatching (dah) (Figure S5).

#### *wt1*

*Wt1* expression in the adult testes of *S. pyrenaicus* (PP) and *S. alburnoides* (AA and PA) was observed in an area where Sertoli cells are located. No clear differences between parental and hybrid samples were observed, but again the gonads of the AA genotype appear to have a more extensive *wt1* expression, possibly in peripheral spermatogonia or interstitial cells (Figure 5g, h and i). Very low levels of *wt1* expression were observed in adult ovaries of hybrid females of *S. alburnoides*, in oocytes at different stages of development (Figure 5j and k).

A faint signal was detected for *wt1* by RT-PCR at early developmental stages. Two distinct bands were obtained from amplification with *wt1* primers using embryos as templates, as opposed to the unique band obtained from adult gonads. Expression in male embryos of AA genotype was observed at 4, 6 and 14dah (Figure S6).

#### *dax1*

*Dax1* expression was observed in adult gonads of both sexes in the *S. alburnoides* complex. Expression in males was seen exclusively outside germ cells in a position compatible with expression in Sertoli cells (Figure 5l). In females of *S. alburnoides*, expression was observed in the adult gonad, in follicle cells surrounding perinuclear stage oocytes (Figure 5m and n), but was absent from cortical alveolar and vitellogenic oocytes.

*Dax1* expression was not observed in early *S. alburnoides* male development at any of the stages analysed (Figure S7).

#### *Figla*

**Figure 6:**
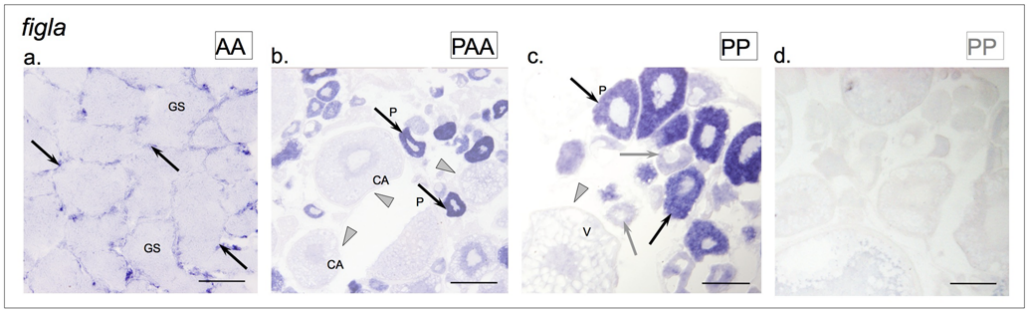
Expression patterns of *figla* in the adult gonads *S. pyrenaicus* and *S. alburnoides*. (a) *S. alburnoides* (AA genotype) testis; (b) *S. alburnoides* (PAA genotype) ovary and *S. pyrenaicus* (PP genotype) ovary with (c) antisense and (d) sense probes (positive signals in primary oocytes exemplified with black arrows; lower expression in early stage oocytes exemplified with grey arrows; cells in different maturation stages, not expressing the transcript are indicated by grey arrowheads). Germ cells (GS), early perinuclear (P), cortical alveolar (CA) and vitellogenic (V) oocytes. Scale bar = 100 µm (a, c, d); scale bar = 200 µm (b).

No sexual dimorphic expression of *figla* was observed in *S. alburnoides*. Amplification of a *figla* specific fragment was conducted successfully both in male and female gonad cDNA templates (Figure S8). Expression was observed in testes in locations compatible with expression in Sertoli cells (Figure 6a). In ovaries *figla* was highly expressed in late primary oocytes, both in bisexual parental species *S. pyrenaicus* and in the *S. alburnoides* hybrids, while only a faint signal was detected in primary oocytes (Figure 6b and c).

## Discussion

In the present study, we report the isolation of four genes known to be involved in the sex determination/gonad differentiation pathways of mammals, predicted to play similar roles in fish, but so far not analysed in the *S. alburnoides* hybrid complex.

*Dmrt1* transcripts were found both in male and female adult gonads. The absence of a sexual dimorphic expression pattern is apparently in contrast to the reported expression patterns for the majority of fish species. In the Nile tilapia *O. niloticus*, *dmrt1* expression occurs exclusively in male gonads [26] and the gene is specifically expressed in male fry very early during development, even before any signs of morphological sex differentiation [61]. In the rainbow trout *O. mykiss*, *dmrt1* is also expressed early in testis development, but not in the ovary [27]. In the more closely related cyprinid zebrafish, though, expression patterns seem to be in agreement to what we report for *S. alburnoides* and *S. pyrenaicus*: *dmrt1* is expressed in the developing gonads of both sexes [28], suggesting that it might play a role in ovary differentiation (as opposed to the classical view of sex dimorphic *dmrt1* expression and an exclusive association with testis development). In fact, evidence from various species suggest that timing and levels of *dmrt1* expression could be associated to sex-specific differentiation fates, with high expression levels correlating with testis development and low *dmrt1* levels being compatible with ovary differentiation [53], [62]–[64]. However, differences emerge when we directly compare the cellular location of *dmrt1* expression in the adult gonads of zebrafish and of *S. alburnoides*. In males of zebrafish, *dmrt1* transcripts were identified in the developing germ cells, while in the *Squalius* samples expression is apparently restricted to Sertoli cell positions. The Sertoli cell lineage specificity and the absence of expression in the germ cell lineage are in accordance with what has been reported for the Nile tilapia [26], Fugu [65] and *dmrt1a* and *dmrt1bY* in the Medaka [66]. Conversely, *dmrt1 expression* in both the Sertoli and the germ cell lineage has been reported in the platyfish *Xiphophorus maculatus* [67]. Thus, the observed differences could be a consequence of different roles in sexual development or differential gene evolution in fish sublineages. The same could apply for gene expression patterns in females. In zebrafish, *dmrt1* gene expression is high in developing perinuclear oocytes and fainter in more mature stages [28], while in *S. alburnoides* females it is mainly observed in the more developed yolk vesicle oocytes.

We have isolated a single *wt1* ortholog in *S. pyrenaicus* in the present work, despite having tested primer combinations based on the two zebrafish genes (*wt1a* and *wt1b*), but we cannot exclude the possibility that a second *wt1* gene is present in these Iberian species. In adult males of *S. alburnoides* and *S. pyrenaicus*, *wt1* is expressed in the Sertoli cell lineage and is apparently absent from germ cells, which is in agreement with what has been reported in other vertebrates [68]. In the medaka, expression of *wt1* genes has been observed in somatic cells during development and in the adult gonads. In adult testis, the location of *wt1a* expression suggested a probable association with germ cell supporting Sertoli cells [34]. Thus, it is possible that *wt1* is playing a similar function in the male gonads of the *Squalius* cyprinids. The apparently lower expression in female gonads could also be related to the role of *wt1* in follicle development. In different mammals and birds [69], higher expression in early stage oocytes and a progressive reduction in *wt1* expression during follicle maturation implied an additional role of this gene: the repression of genes involved in follicle differentiation, and the maintenance of a number of oocytes that can later on be recruited, in early developmental stages. It is not clear whether or not this is the role that *wt1* is playing in *S. alburnoides* ovary but the fact that most oocytes present in the analysed samples are of later stages of maturation and have low *wt1* expression is consistent with that possibility.

The non-dimorphic expression pattern exhibited by *dax1* in gonads of adult *Squalius* is in accordance with what has been described in a number of other teleost species. Non sex specific expression between female and male adult gonads was found in the nile tilapia *O. niloticus* [56] and throughout the gonadal sex differentiation period in the European sea bass *D. labrax* [57]. Conversely, in medaka, *dax1* mRNA was absent from the adult testis, but expression was detected in postvitellogenic follicles of the adult ovary [58]. The expression pattern of this gene in the *Squalius* gonads, restricted to Sertoli cells in males and granulosa cells in females, could well correlate with the functions of modulation of the activity of other factors and participation in the maintenance of testis and ovary structure and organization [50]. In fact, it has been proposed that the effects observed in mouse Dax1 mutants, which affect normal granulosa cell organization around oocytes in females, could be functionally related to the defects in Sertoli cell support of germ cells observed in males [70]. Taking into account the expression patterns of *dax1* in the same cell types in *Squalius*, it is possible that in the adult gonads of both sexes, *dax1* could also be contributing to the regulation of these somatic cell lineages and thus indirectly affecting gametogenesis.

For all genes, an initial assessment was performed of whether, besides their contribution to regulation in the adult gonads, they might also have a direct role in the earlier process of sex determination. The evaluation of a putative role and thus interest for further analysis would be based on whether they were expressed early during development in the developing gonadal structures. As no sex marker is available for the *Squalius* species, we selected the putative all-male progeny of AA genotype to perform the test, avoiding the ambiguities that could result from the use of a diversity of individuals of unknown sex. We observed that, in agreement with other teleosts [27], [40], [61], both *dmrt1* and *wt1* are expressed in *S. alburnoides* male progeny, at early stages and in the developing gonad, suggesting a contribution to its differentiation. However, the real participation of *wt1* and *dmrt1* as sex determination genes can only be assessed when male and female embryos can be analysed in parallel. The same is true for *dax1* expression. No *dax1* transcripts were observed in the early developmental stages of *S. alburnoides* males. It is possible that levels of *dax1* expression might vary during development, as shown in mouse [71] and that the stages analysed in *S. alburnoides* could correspond to “windows” of *dax1* downregulation. However, the absence of *dax1* expression at early developmental stages argues against its involvement in male sex determination, without excluding the possibility of a later role in gonad differentiation.

Regarding *figla*, and its potential use as an early ovary marker in *Squalius*, the expression results obtained in adult gonads indicate a completely opposite scenario as initially predicted. Female restricted *figla* expression has been described for a number of species, namely in mammals and fish [60], [73], so it was unexpected to isolate a *figla* transcript from *S. pyrenaicus* and *S. alburnoides* adult testis. No differences were found between female and male *figla* isolates in terms of sequence, domain prediction and putative functionality.

In fish like the gilthead seabream *Sparus aurata*, sex change occurs at a certain stage of the life cycle, from a heterosexual gonad, with gene expression profiles changing accordingly [73]. Thus, an initial hypothesis would be that gonad maturation in *S. alburnoides* could also involve a similar process and that *figla* transcripts detected by RT-PCR could originate from a vestigial female structure. Data from *in situ* hybridisation did not support this hypothesis. *Figla* transcripts were detected in Sertoli cell locations in males with normal testicular organization and with no morphological indication of the presence of any female specific gonadal tissues. In females, on the other hand, *figla* expression apparently fits the expectations. Expression was restricted to cells surrounding early stage oocytes, which is in close agreement with what has been reported in the medaka [74] and consistent with the role of *figla* in follicle development. A more reasonable possibility to account for non-dimorphic expression would be that *figla* transcripts would occur both in *Squalius* males and females and differential expression levels of *figla* during development would promote male or female differentiation. In fact, a peak in *figla* expression has been correlated with ovary differentiation in zebrafish [72]. An alternative hypothesis would be that *figla* female specific expression would only occur during development and that in the adult it would be expressed in cells supporting the germ cell lineage. These possibilities can only be tested more systematically when a straightforward identification of male and female *Squalius* embryos becomes possible and the expression patterns can be followed in parallel in both sexes. Additionally, it would be of interest to follow expression of *figla* and of the other sex determination candidates quantitatively, at different stages of gonad development, as timing and expression levels of genes in sex determination cascades have been shown to be critical to promote the switch between male and female determination pathways [62]–[65], [73].

A more comprehensive analysis of the putative roles and interactions of the isolated candidate genes is currently hampered by the impossibility of unambiguous sexing of embryos. While trying to overcome this difficulty, our aim was to create the framework that could subsequently be extended in terms of further analysis. No significant differences were found regarding the cellular location and the expression patterns of individual genes in gonads of the parental species *S. pyrenaicus* and in the hybrid *S. alburnoides*, even in an heterogeneous context of germ cell development, implying that, at least for the set of genes analysed, the normal patterns of gene expression are being resumed even upon hybridisation and that the normal process of cyclical differentiation and maintenance of gonad identity is taking place in hybrids as in parental species. Globally, the gene expression patterns observed in *S. alburnoides* are apparently in accordance with what has been described for other teleost species, implying that there is a good conservation of players and functions that might “resist” genome remodeling events such as hybridisation. Therefore, rather than a definitive answer, the present work has provided the first clues and the initial working basis that can be expanded in the attempt to elucidate a gene-mediated sex determination in *S. alburnoides*. This then may contribute to a broader understanding of the complex and diverse pathways of sex determination in fish.

## Materials and Methods

### Samples

The specimens used in this study were collected from the Tejo River Basin (River Raia), as previously described [15]. A total of 9 gonad samples, 3 *of S. pyrenaicus* and 6 of *S. alburnoides* (2 nuclear non-hybrid, 2 diploid, and 2 triploid hybrids) were selected from the global sample already analysed and genotyped using microsatellites [15]. An additional *S. pyrenaicus* sample was included and processed as described in [15] (a summary of the selected samples is presented in Table 2-a).

### RNA extraction and cDNA synthesis

Total RNA was extracted from adult gonads of *S. pyrenaicus* and *S. alburnoides* using the TRIZOL reagent (Gibco-BRL) according to the supplier's recommendation. First strand cDNA was synthesized with RevertAid™ First Strand cDNA Synthesis Kit (Fermentas), using random hexamer primer.

### Isolation of *dmrt1*, *wt1*, *dax1* and *figla*

A partial fragment of the *dmrt1* gene of *S. pyrenaicus* (PP) and *S. alburnoides* (AA) was amplified using degenerate and specific primers (Table 1), according to conditions previously described [75]. To obtain initial sequence information for the isolation of the *wt1* gene, degenerate primers (Table 1 – WT1 F1a; WT1 R1a; WT1 F1b; WT1 R1b; WT1 F5) were designed based on coding sequences of this gene from *D. rerio* (NM_131046; NM_001039634), *A. japonica* (AB030741) and *O. mykiss* (AF334670; AF334671). Amplification of a partial sequence of the *dax1* gene of *S. pyrenaicus* and *S. alburnoides* was performed using primers based on zebrafish *dax1* (ENSDART00000020212) (Table 1). Primers for the *Figla* gene were designed based on zebrafish sequences (NM_198919) (Table 1). All primers were tested on cDNA samples of *S. pyrenaicus* (PP) and *S. alburnoides* (AA and PAA) gonads, according to the following PCR conditions: pre-heating at 96°C for 2 min 30 s, 35 cycles at 96°C for 30 s, 54°C or 56°C (*dax1*) for 45 s and 72°C for 1 min 15 s and a final extension at 72°C for 10 min. Products corresponding to the A and P genomes were sequenced and analysed using Sequencher ver. 4.0 (Gene Codes Corporation, Inc.).

### Cloning

Amplification products of the four genes were cloned into pDrive Cloning Vector (Qiagen), positive colonies were identified by blue/white selection and screened with M13F and M13R universal primers. Positive colonies were randomly picked and sequenced. Sequences were analysed using Sequencher ver. 4.0 (Gene Codes Corporation, Inc.) and compared with sequences corresponding to the P and A genomes, previously isolated from *S. pyrenaicus* (PP) and *S. alburnoides* nuclear non-hybrid (AA) males.

### Protein alignment and phylogenetic analysis

The deduced amino acid sequence of the *S. pyrenaicus* (PP-DMRT1) and *S. alburnoides* (AA-DMRT1) *dmrt1* was aligned with *D. rerio* (Dre-DMRT1, AAQ04555) *Takifugu rubripes* (Tru-DMRT1, BAE16952), *O. mykiss* (Omy-DMRT1, AAG17544) and *O. latipes* (Ola-DMRT1, AAL02165). The deduced amino acid sequence of the *S. pyrenaicus* (PP-WT1) *wt1* ortholog was aligned with *D. rerio* (Dre-a-WT1, NP_571121; Dre-b-WT1, NP_001034723), *O. mykiss* (Omy-a-WT1, AAK52719; Omy-b-WT1, AAK52721), *A. japonica* (Aja-WT1, BAA90558) and *O. latipes* (Ola-WT1, BAC10628). Alignments were also performed for the deduced amino acid sequences of the two remaining genes. For *dax1*, aminoacid sequences of *D. rerio* (Dre-DAX, Q1L693), *Dicentrarchus labrax* (Dla-DAX1, CAG17628), *O. latipes* (Ola-DAX1, NP_001104259) and *O. niloticus* (Ony-DAX1, AAN17672) were aligned with the *putative S. pyrenaicus* (PP-DAX1) and *S. alburnoides* (AA-DAX1) orthologs. The deduced amino acid sequence of *Figla* of *S. pyrenaicus* (PP_FiglaM and PP_FiglaF – from male and female samples) and *S. alburnoides* (from male samples AA- FiglaM) were aligned with *D. rerio* (Dre-FIGLA, NP_944601), *Tetraodon nigroviridis* (Tni-FIGLA, ACH91670) and *Kryptolebias marmoratus* (Kma-FIGLA, ABG89136).

Specific protein domains for the Iberian species were identified by homology search using Simple Modular Architecture Research Tool (SMART) [76]. For the phylogenetic reconstruction, both amino acid sequences of DMRT1 from other species and other proteins sharing the DM DNA-binding domain were additionally included: *Homo sapiens* DMRT1 (Hsa-DMRT1, CAB99335), *Gallus gallus* DMRT1 (Gga-DMRT1, Q9PTQ7), *H. sapiens* DMRT2 (DMRT2-Hsa, AL358976), *O. latipes* DMRT2 (DMRT2-Ola, AAL02163), *H. sapiens* DMRT3 (Hsa-DMRT3, CAB99336), *T. rubripes* DMRT3 (DMRT3-Tru, BAE16954) and *D. rerio* DMRT3 (DMRT3-Dre, AAU89440). For WT1, in addition to the sequences used in the protein alignments, amino acid sequences from orthologs from other species were also used: *H. sapiens* WT1 (Hsa-WT1, CAI95759), *G. gallus* WT1 (Gga-WT1, NP_990547), *Mus musculus* WT1 (Mmu-WT1, P22561) and *Rattus norvegicus* WT1 (Rno-WT1, NP_113722). For DAX1, phylogenetic reconstruction was based on the teleost and other species DAX1 proteins as well as other protein family members, including: *H. sapiens* DAX1 (Hsa-DAX1, AAC13875), *M. musculus* DAX1 (Mmu-DAX1, NP_031456), *G. gallus* (Gga-DAX1, NP_989924) and *O. niloticus* DAX2 (Oni-DAX2, ABB88832). For FIGLA, phylogenetic relationships were inferred based on the teleost sequences already used in the alignment and FIGLA orthologs from other species: *H. sapiens* FIGLA (Hsa- FIGLA, AAS48452), *R. norvegicus* FIGLA (Rno-FIGLA, XP_575589) and *Xenopus (Silurana) tropicalis* FIGLA (Xtr FIGLA, NP_001016342). Phylogenetic trees were constructed using the Neighbour-Joining method as implemented in MEGA version 2.1 [77]. Support values for the observed topologies were generated by 1000 bootstrap replicates.

### Protein structure comparison

Secondary structure was predicted, based on the amino acid sequences of the *dmrt1* gene fragments of *S. pyrenaicus* and *S. alburnoides* using the PSIPRED method [78] as implemented in PSIPRED server [79].

### Analysis of candidate gene expression in adult gonads of *S. pyrenaicus* and *S. alburnoides*

#### Probe synthesis

Colonies selected after the cloning procedure (representing the P or A genomes) were cultured overnight and purified with aMiniprep Kit (Invitrogen). Sense and antisense DIG-labelled riboprobes were obtained by digestion with *Hind*III or *Bam*HI (in the case of *dmrt1*, *wt1* and *figla*), *Hind*III and *Kpn*I (*dax1*) and transcription with SP6 and T7 polymerases (DIG RNA Labelling Kit - Roche).

#### Tissue preparation and sectioning and *in situ* hybridisation

Testis from adult males of *S. pyrenaicus* and *S. alburnoides* (AA and PA) and ovaries of diploid (PA) and triploid (PAA) females of *S. alburnoides* were fixed and processed according to [80]. Embedded gonads were then frozen and stored at −80°C. Cryosections of 10 µm were obtained and collected on Superfrost Plus slides (VWR). *In situ* hybridisation was performed as previously described [15].

#### Collection of embryos and *in situ* hybridisation

Embryos of different developmental stages and of specific genotype (diploid AA) were selected from the progeny of the experimental Cross I (see [15] for the cross and Table 2-b for information about the selected progeny). In the absence of a molecular sex marker to unambiguously identify sexes in the *S. alburnoides* complex we relied on the extremely strong correlation between AA genotype and male phenotype, with only one report of a single female individual of AA genotype in 20 years of intensive study of the complex [81]. Thus, a group of samples was selected, for which sex could be inferred (in this case male sex) with a high degree of confidence. Progeny had been fixed, processed, frozen and stored at −80°C according to [80]. Cryosections of 14 µm were obtained and collected on Superfrost Plus slides (VWR). *In situ* hybridisation was performed as previously described [15].

## Supporting Information

Figure S1 Protein alignment of *Squalius* Dmrt1 with Dmrt1 orthologs of other teleosts. (*) Identical residues in all sequences in the alignment; (:) Conserved substitutions; (.) Semi-conserved substitutions. The conserved DM domain that characterizes the family is highlighted in grey. ▴ *S. pyrenaicus* (PP); • *S. alburnoides* (AA)
(1.69 MB TIF)

Figure S2 Protein alignment of *S. pyrenaicus* Wt1 with Wt1 orthologs of other teleosts. (*) Identical residues in all sequences in the alignment; (:) Conserved substitutions; (.) Semi-conserved-substitutions. The zinc finger and WT1 specific domains that characterize the protein are highlighted in grey. ▴ *S. pyrenaicus* (PP)
(2.49 MB TIF)

Figure S3 Protein alignment of the *Squalius* Dax1 with Dax1 orthologs of other teleosts. (*) Identical residues in all sequences in the alignment; (:) Conserved substitutions; (.) Semi-conserved-substitutions. The LXXL motifs and the ligand binding domain that characterize Dax1 are highlighted in grey. ▴ *S. pyrenaicus* (PP); • *S. alburnoides* (AA)
(2.16 MB TIF)

Figure S4 Protein alignment of the *Squalius* Figla with Figla orthologs of other teleosts. (*) Identical residues in all sequences in the alignment; (:) Conserved substitutions; (.) Semi-conserved-substitutions. The helix-loop-helix domain, characteristic of the Figla proteins is highlighted in grey. Amino acid sequences obtained from ▴ *S. pyrenaicus* males (PP-M); Δ females (PP-F)and • *S. alburnoides* (AA).
(1.32 MB TIF)

Figure S5 Expression of *dmrt1* during *S. alburnoides* development. (a) RT-PCR analysis of *dmrt1* in embryos at 2, 6 and 12 days after hatching (dah); (b) Location of dmrt1 expression in an embryo section at 6dah in the presumptive location of the developing gonad (arrow). Spinal cord (SC); gut (G); yolk sac (Y). Scale bar = 100 µm.
(1.42 MB TIF)

Figure S6 Expression of *wt1* during *S. alburnoides* development. (a) RT-PCR analysis of *wt1* in embryos at 2, 4, 6 and 14 days after hatching (dah); (b) Location of *wt1* expression in embryo sections at 6dah and 12dah in the presumptive location of the developing gonad (arrow). Mesoderm (M); gut (G); yolk sac (Y). Scale bar = 100 µm.
(2.67 MB TIF)

Figure S7 Absence of *dax1* positive signals in embryo sections of *S. alburnoides* (AA genotype). In situ hybridisation at 6 (sense probe in grey) and 12 days after hatching (dah). Spinal cord (SC); gut (G); yolk sac (Y). Scale bar = 100 µm.
(3.35 MB TIF)

Figure S8 RT-PCR analysis of figla in adult gonad tissue. *S. pyrenaicus* (PP) females; *S. alburnoides females* (PAA) and males (AA). Actin controls for the same samples are shown in the lower row.
(0.38 MB TIF)
