# Supplementary figures and images for: Sex Determination in the Squalius alburnoides Complex: An Initial Characterization of Sex Cascade Elements in the Context of a Hybrid Polyploid Genome
Source: PLoS One. 2009 Jul 28;4(7):e6401. doi: 10.1371/journal.pone.0006401 (PMC2713423; doi:10.1371/journal.pone.0006401)

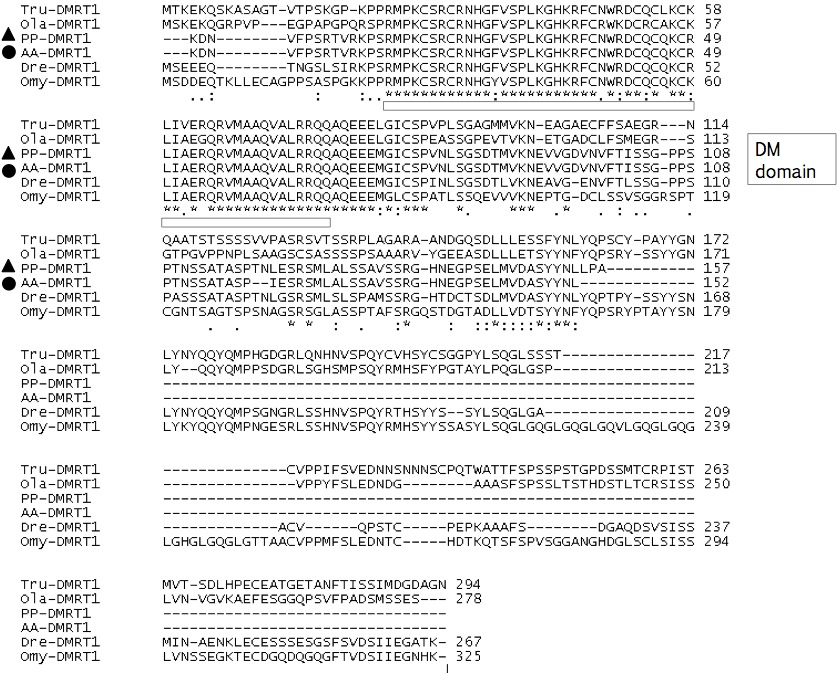

Supplement: Figure S1 — Protein alignment of Squalius Dmrt1 with Dmrt1 orthologs of other teleosts. (*) Identical residues in all sequences in the alignment; (:) Conserved substitutions; (.) Semi-conserved substitutions. The conserved DM domain that characterizes the family is highlighted in grey. ▴ S. pyrenaicus (PP); • S. alburnoides (AA) (1.69 MB TIF) [file pone.0006401.s001.tif]

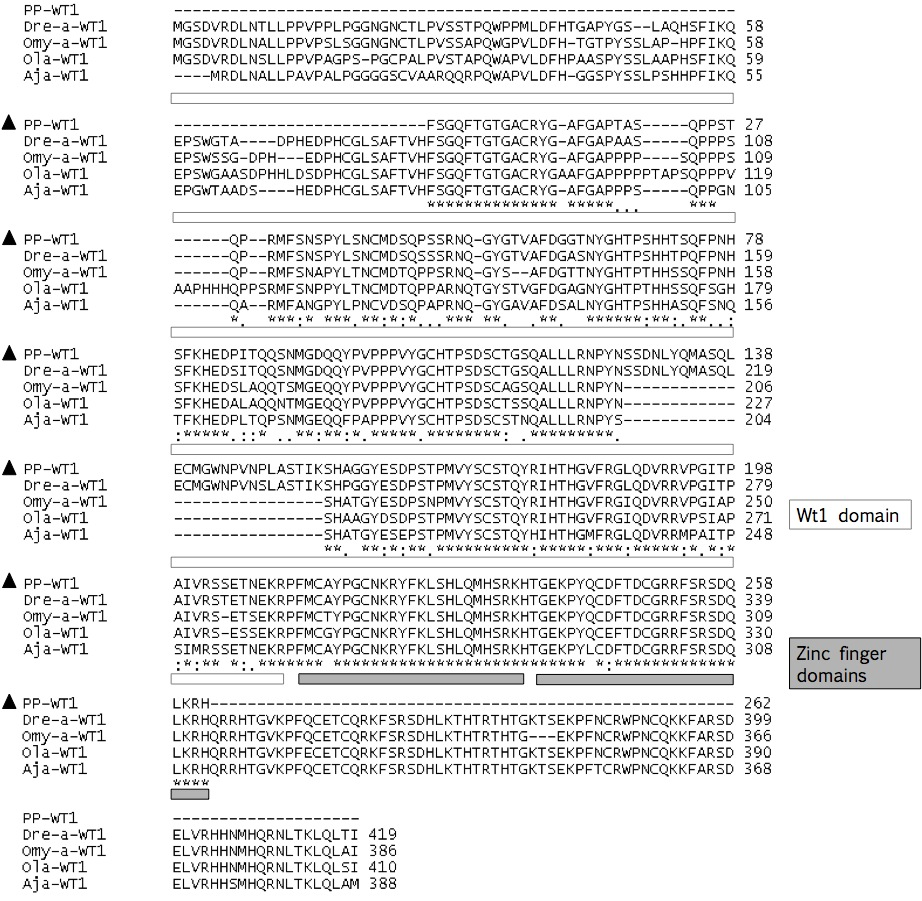

Supplement: Figure S2 — Protein alignment of S. pyrenaicus Wt1 with Wt1 orthologs of other teleosts. (*) Identical residues in all sequences in the alignment; (:) Conserved substitutions; (.) Semi-conserved-substitutions. The zinc finger and WT1 specific domains that characterize the protein are highlighted in grey. ▴ S. pyrenaicus (PP) (2.49 MB TIF) [file pone.0006401.s002.tif]

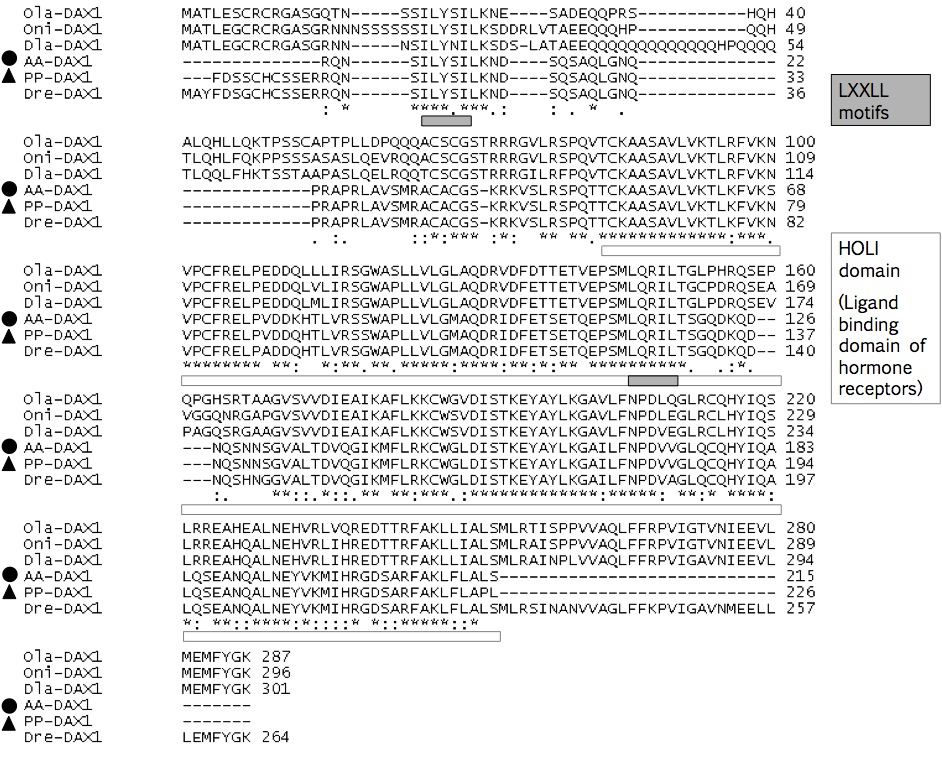

Supplement: Figure S3 — Protein alignment of the Squalius Dax1 with Dax1 orthologs of other teleosts. (*) Identical residues in all sequences in the alignment; (:) Conserved substitutions; (.) Semi-conserved-substitutions. The LXXL motifs and the ligand binding domain that characterize Dax1 are highlighted in grey. ▴ S. pyrenaicus (PP); • S. alburnoides (AA) (2.16 MB TIF) [file pone.0006401.s003.tif]

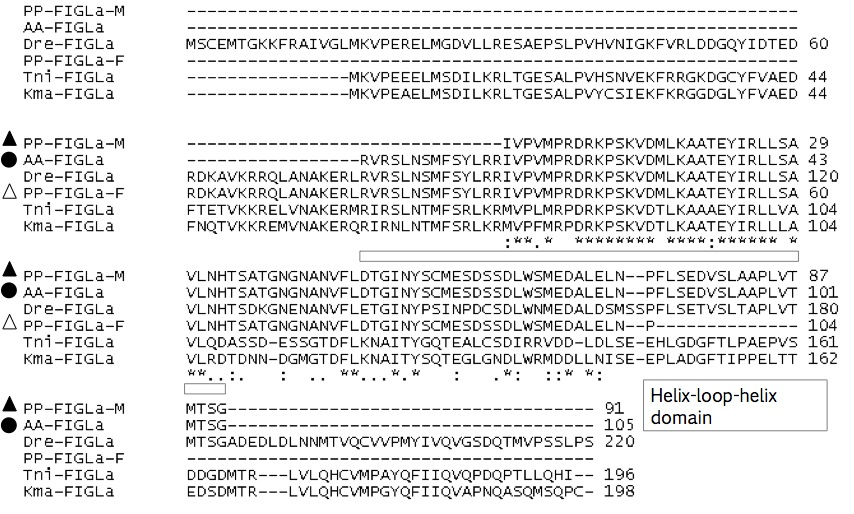

Supplement: Figure S4 — Protein alignment of the Squalius Figla with Figla orthologs of other teleosts. (*) Identical residues in all sequences in the alignment; (:) Conserved substitutions; (.) Semi-conserved-substitutions. The helix-loop-helix domain, characteristic of the Figla proteins is highlighted in grey. Amino acid sequences obtained from ▴ S. pyrenaicus males (PP-M); Δ females (PP-F)and • S. alburnoides (AA). (1.32 MB TIF) [file pone.0006401.s004.tif]

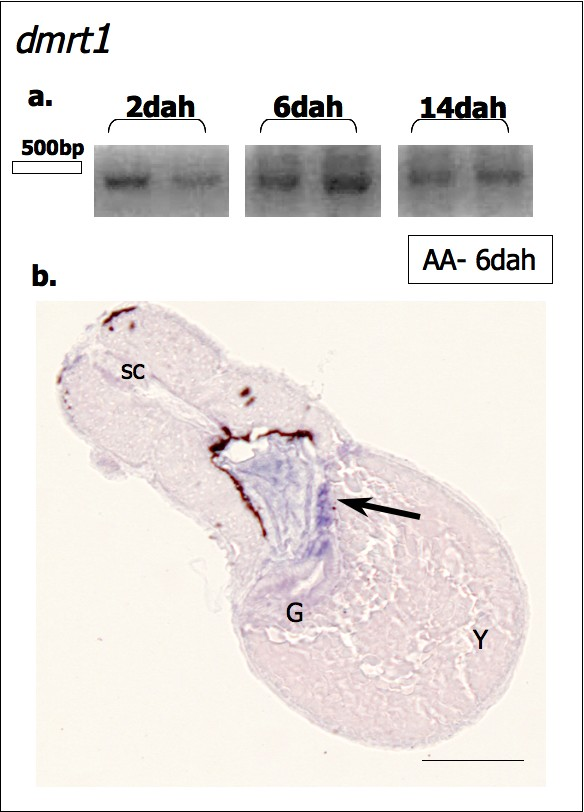

Supplement: Figure S5 — Expression of dmrt1 during S. alburnoides development. (a) RT-PCR analysis of dmrt1 in embryos at 2, 6 and 12 days after hatching (dah); (b) Location of dmrt1 expression in an embryo section at 6dah in the presumptive location of the developing gonad (arrow). Spinal cord (SC); gut (G); yolk sac (Y). Scale bar = 100 µm. (1.42 MB TIF) [file pone.0006401.s005.tif]

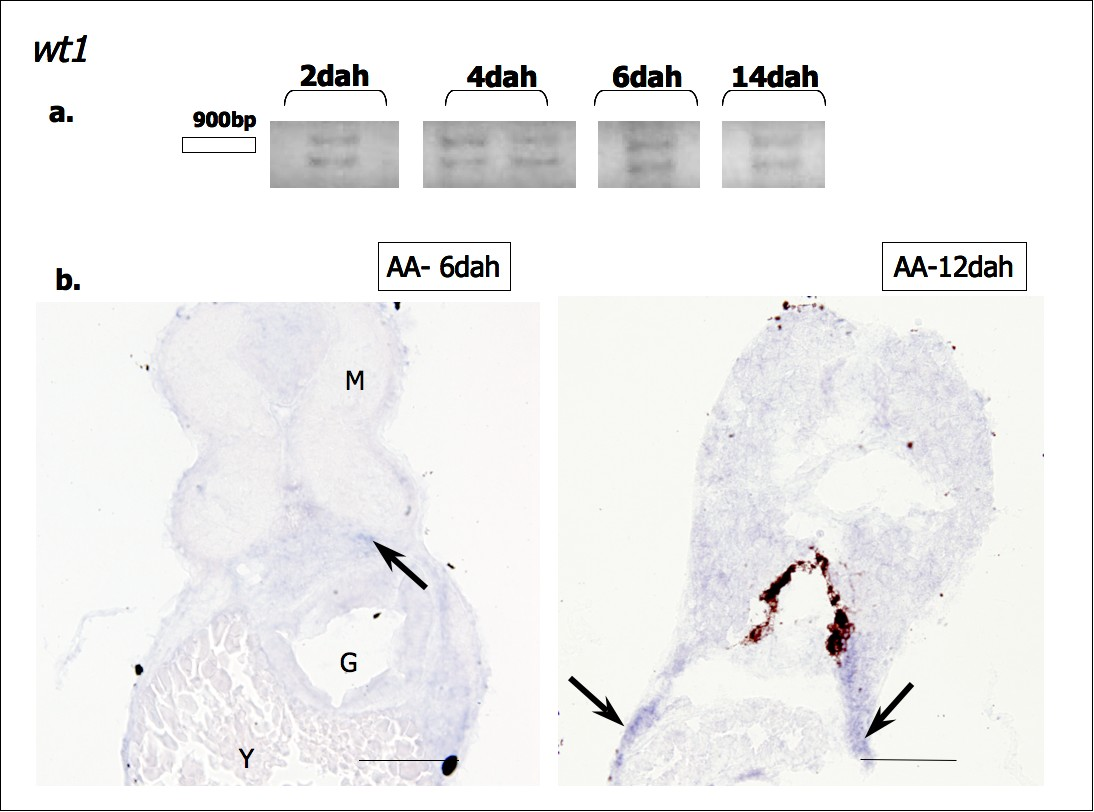

Supplement: Figure S6 — Expression of wt1 during S. alburnoides development. (a) RT-PCR analysis of wt1 in embryos at 2, 4, 6 and 14 days after hatching (dah); (b) Location of wt1 expression in embryo sections at 6dah and 12dah in the presumptive location of the developing gonad (arrow). Mesoderm (M); gut (G); yolk sac (Y). Scale bar = 100 µm. (2.67 MB TIF) [file pone.0006401.s006.tif]

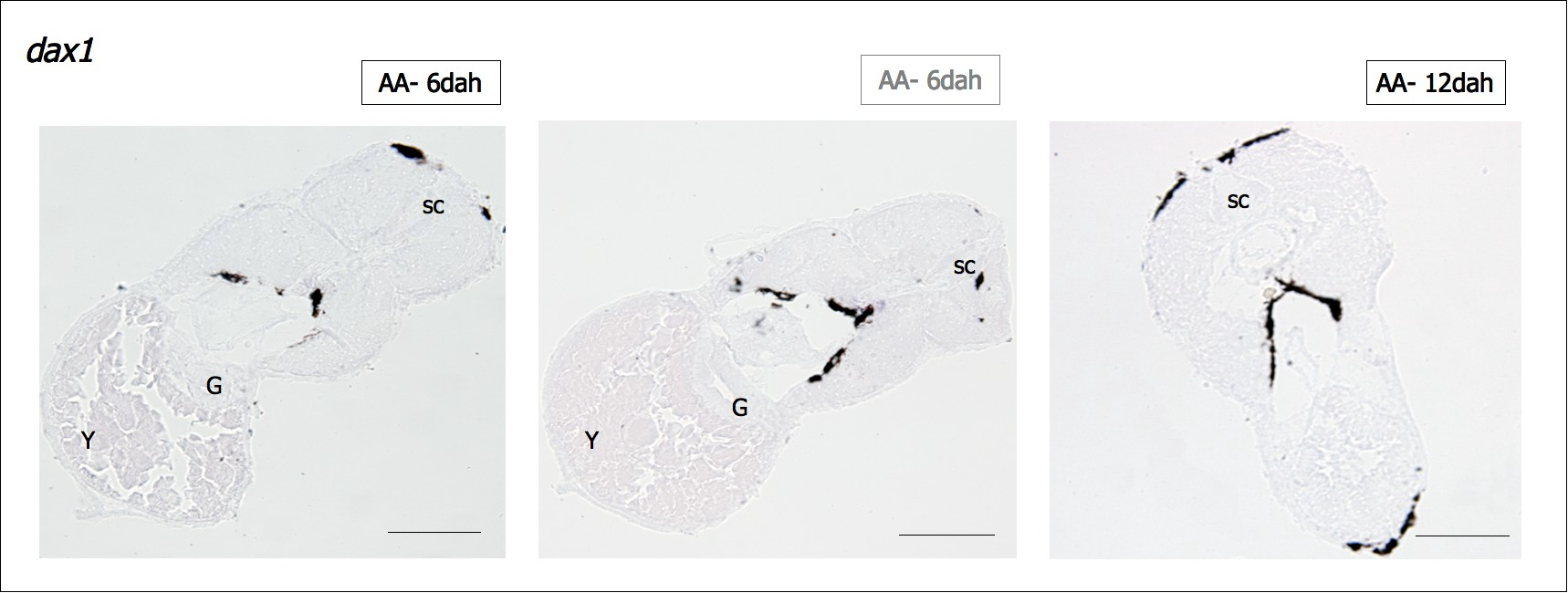

Supplement: Figure S7 — Absence of dax1 positive signals in embryo sections of S. alburnoides (AA genotype). In situ hybridisation at 6 (sense probe in grey) and 12 days after hatching (dah). Spinal cord (SC); gut (G); yolk sac (Y). Scale bar = 100 µm. (3.35 MB TIF) [file pone.0006401.s007.tif]

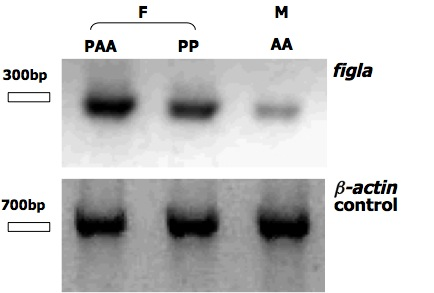

Supplement: Figure S8 — RT-PCR analysis of figla in adult gonad tissue. S. pyrenaicus (PP) females; S. alburnoides females (PAA) and males (AA). Actin controls for the same samples are shown in the lower row. (0.38 MB TIF) [file pone.0006401.s008.tif]
